# TROIKA‐1: A double‐blind, randomized, parallel group, study aimed to demonstrate the equivalent pharmacokinetic profile of HD201, a potential biosimilar candidate to trastuzumab, versus EU‐Herceptin^®^ and US‐Herceptin^®^ in healthy male subjects

**DOI:** 10.1002/prp2.839

**Published:** 2021-07-26

**Authors:** Martin Demarchi, Pierre Coliat, Kristi Mclendon, Jocelyn Chung Shii Hii, Peggy Feyaerts, Felicia Ang, Litha Jaison, Filip Deforce, Marie Paule Derde, Michael Jinwoo Kim, Lisa Soyeon Park, Alexandre Detappe, Xavier Pivot

**Affiliations:** ^1^ Institute of Cancer Strasbourg (ICANS) Strasbourg France; ^2^ Royal Brisbane and Women’s Hospital Brisbane Australia; ^3^ Prestige Biopharma Ltd Singapore Singapore; ^4^ DICE NV Brusseles Belgium; ^5^ Prestige Biologics Co Ltd Seoul Korea; ^6^ Paul Strauss Cancer Center Strasbourg France

**Keywords:** biosimilar, pharmacokinetic, trastuzumab

## Abstract

Prestige Biopharma Ltd (Singapore) has developed HD201, a proposed biosimilar to reference product trastuzumab. As a part of the stepwise approach to ensure comparability between the biosimilar candidate and the reference medicinal product, a phase I study in healthy subjects was conducted to demonstrate the pharmacokinetic (PK) equivalence (NCT03776240). The primary objective of the study was to demonstrate (PK) equivalence of HD201, EU‐Herceptin^®^, and US‐Herceptin^®^ given at 6 mg/kg as a 90‐min i.v. infusion to healthy male subjects. A pairwise comparisons based on the primary endpoint AUC_0–inf_ and secondary PK endpoints, AUC_0–last_ and *C*_max_ were undertaken. PK equivalence was to be concluded if the 90% confidence interval (CI) for the ratio of geometric means for each criterion were within the equivalence margin of 80% to 125%. Secondary objectives included assessment of other PK parameters, safety, tolerability, and immunogenicity in the three arms. A total of 105 healthy male subjects (35/treatment) were randomized in this study. The 90% CI for the ratios of AUC_0–inf_, *C*_max_ and AUC_0–last_, were within 80%–125% for the comparisons of HD201 to EU‐Herceptin^®^ or US‐Herceptin^®^ and EU‐Herceptin^®^ to US‐Herceptin^®^. The frequency of subjects with TEAEs of special interest was slightly lower in the HD201 group (20.0%) compared to the other treatment groups (EU‐Herceptin^®^: 34.3%; US‐Herceptin^®^: 31.4%). Only 1 subject (EU‐Herceptin^®^ group) developed anti‐drug antibodies prior to dosing. Overall, HD201 demonstrates PK similarity to both EU‐Herceptin^®^ and US‐Herceptin^®^. The three study drugs also demonstrated similar safety profiles.

## Graphical abstract

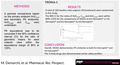

**Abbreviations**
ADA: anti‐drug antibody
ANOVA: analysis of variance
CI: confidence interval
ECHO: echocardiogram
EMA: European Medicines Agency
FDA: Food and Drug Administration
HER2: human epidermal growth factor Receptor 2
HRP: horseradish peroxidase
PK: pharmacokinetic

## 1 INTRODUCTION

Trastuzumab containing regimens are the backbone for the treatment of human epidermal growth factor Receptor 2 (HER2)‐positive breast cancer, providing significant clinical benefit for metastatic breast cancer and increasing the proportion of cured patients in the adjuvant setting for early breast cancer.^1^, ^2^ As the patents on the licensed trastuzumab expired, there has been an increasing interest in developing biosimilar as the approval of biosimilar can facilitate patient access to high‐quality biologic medicine at a lower cost. Prestige Biopharma Ltd. (Singapore) has developed HD201, a potential biosimilar to reference product trastuzumab. The European Medicines Agency (EMA) and the US Food and Drug Administration (FDA) have developed specific guidelines for a biologic drug to be approved as a biosimilar.^3^, ^4^ Among the stepwise approach to ensure comparability between the biosimilar candidate and the reference medical product, a phase I study in healthy subjects is recommended to demonstrate the pharmacokinetic (PK) equivalence. EAGLE‐I‐12, a first phase I study demonstrated PK equivalence between HD201 and European Union sourced Herceptin^®^ (EU‐Herceptin^®^) (EudraCT number 2012‐000805‐56).^5^ Since, to ensure the production of the biologics at an industrial scale, some changes have been introduced in the production process of HD201. These changes, induced the need to repeat the overall comparability exercise. A new phase I study (TROIKA‐1) in healthy subjects was designed with at the request of the US regulatory agency (US FDA) the addition of an US‐sourced Herceptin^®^. In this study (NCT03776240), pairwise comparisons were conducted between HD201 and both US‐sourced and European‐sourced Herceptin.

## 2 MATERIALS AND METHODS

### 2.1 Study design

This was a single center, phase I, double‐blind, unstratified, randomized, single‐dose, three‐arm parallel group study in healthy adult male subjects. The primary objective of the study was to demonstrate PK equivalence of HD201 (Prestige Biopharma. Ltd, Singapore), EU‐Herceptin^®^, (Roche Pharma AG), and US‐Herceptin^®^ (Genentech). Secondary objectives included assessment of other PK parameters, safety, tolerability, and immunogenicity in the three arms.

For inclusion into the study, subjects had to be between the ages of 18 and 55 years. All subjects had to have normal screening results for vital signs, physical examination, and hematologic, renal, and hepatic functions. A normal 12‐lead ECG and a left ventricular ejection fraction (LVEF) >60% according to echocardiogram (ECHO) were required. Subjects who had a history of cardiac disease, cancer, or any clinically significant disease were excluded.

Eligible subjects were randomized 1:1:1, to receive a single dose (6 mg/kg) of either HD201, EU‐Herceptin^®^, or US‐Herceptin^®^ by intravenous infusion over 90 min.

This double‐blinded randomized study was conducted in compliance with Good Clinical Practice and the Declaration of Helsinki. The study protocol and its amendments were approved by the Independent Ethics Committee of Australia, and all of the participants provided written informed consent prior to initiation of any study‐related procedures.

Subjects were confined from at least 10 h before dosing until after the 48‐h post‐dose blood draw. The duration of the study for each subject was approximately 14 weeks: 4 weeks for screening, and 8 weeks for single‐dose PK and safety assessments. The PKC population included the cases with PK samples collection even if not enough samples were available to estimate all PK parameters. PKP population included cases with all PK parameters estimated.

### 2.2 Pharmacokinetic assessments

An immunoassay method was validated for the determination of HD201 or Herceptin^®^ in human serum using an ELISA method. This ELISA was designed to quantify HD201 or Herceptin^®^ in human serum. Plates were coated with anti‐trastuzumab antibody and then blocked to minimize any non‐specific binding. Diluted human serum samples were then added and the plate was incubated. Subsequently, plates were washed and any Trastuzumab present in the samples were bound by the immobilized antibody and unbound substances were washed away. Analyte was detected by subsequent addition of mouse anti‐human IgG (Fc) CH2 domain antibody labeled with horseradish peroxidase (HRP). A colorimetric signal was generated by further addition of Tetramethylbenzidine (TMB) substrate and stop solution. Plates were read in a microplate reader at 450 nm with a reference wavelength of 620 nm. The signal produced was proportional to the amount of analyte present and it was interpolated from the calibration curve using a four‐parameter logistic curve‐fitting program. Experiments were carried out to determine bioanalytical parallelism in the ligand‐binding assay. Minimum required dilution, dilutional integrity, selectivity, and specificity in normal male serum and matrix effects (healthy and diseased populations) were considered in the PK validation assay.

The method was successfully validated over the calibration range 1.00–100 μg/ml (LLOQ: 2.00 μg/ml, ULOQ: 70.00 μg/ml) and precision and accuracy for all validation parameters passed the acceptance criteria.

A total of 13 blood samples at predefined timepoints were drawn from each subject for PK analyses. Blood samples were collected prior to drug administration, 1.5 and 3 h, (from the start of infusion), 8, 24, 48, 96, 168, 336, 504, 672, 1008, and 1272 h. Blood samples for PK analysis or immunogenicity were centrifuged at 1300 to 2000 g for 10 min at 20℃. Two aliquots of atleast 0.5‐ml serum were transferred to appropriate tubes and stored at approximately –80℃ until the sample analysis for PK. and immunogenicity. PK and ADA analysis was performed by the Agilex Biolabs (28 Dalgleish Street). NAb analysis was not performed for this study as the positive ADA was from the pre‐dose sample thus it is concluded as unrelated to the treatment. These bioanalyses were performed in compliance with the GCP and also in accordance with the current regulations as per the industry standards: Guidelines on Bioanalytical Method Validation, Good Laboratory Practices (GLP) and Guideline for GCP ICH E6.

### 2.3 Pharmacokinetic parameters

The primary endpoint for this study was AUC_0–inf_ (the area under the concentration–time curve from the initial time point, extrapolated to infinity), *C*_max_ (maximal concentration), AUC_0–last_ (the area under the concentration–time curve from the initial time point, extrapolated to last detected dosage). PK equivalence of HD201 to each reference product was to be concluded if the 90% confidence interval for the ratio of geometric means for each criterion were within the conventional equivalence margin of 80% to 125%.

AUC was calculated by using the linear trapezoidal rule, with actual elapsed time values. The volume of distribution (*V*_d_), *C*_max_, and *t*_max_ were obtained directly from the observations. *K*_el_ is the negative of the estimated slope of the linear regression of the log‐transformed concentration (natural logarithm) versus time profile in the terminal elimination phase. At least three concentration points were used in estimating *K*_el_. The half‐life (*t*_1/2_) was calculated as ln (2)/*K*_el_. Total clearance was calculated as dose/AUC_0–∞_, and *V*_d_ was calculated as dose/*K*_el_ × AUC_0–∞_.

### 2.4 Statistical analysis

Pharmacokinetic analysis was performed using Phoenix^®^ WinNonlin^®^, which is validated for bioequivalence/bioavailability studies. Inferential statistical analyses were performed using SAS^®^ according to EMA and FDA guidelines.

Analysis of variance (ANOVA) was applied to natural log‐transformed data for AUC_0–inf,_ AUC_0–last_, and *C*_max_. Treatment was incorporated in the model as a fixed factor with three levels. The normal distribution of values was assessed and based on least‐squares means from the ANOVA, the geometric mean and 95% confidence interval (CI) for each treatment, and the ratio of geometric means and 90% CI for the ratio of geometric mean were calculated. These were presented after back‐transformation to the original scale. As foreseen in the protocol and the Statistical Analysis Plan, no correction for multiplicity was applied in the analysis.

Pairwise comparisons were performed between HD201 and US‐Herceptin^®^ groups, HD201 and EU‐Herceptin^®^ groups and US‐Herceptin^®^ and EU‐Herceptin^®^ groups. Bioequivalence (similarity) was achieved if 90% CI of the ratio of geometric means of log‐transformed values, based on least‐squares means from the ANOVA, were included within the interval 80.00% to 125.00%.

Based on literature and sponsor data, inter‐subject coefficient of variation was estimated to be 17% and 19% for AUC and *C*_max_, respectively. With those expected coefficients of variation, assuming a ratio of AUC and *C*_max_ between 0.925 and 1.08 and a power of at least 85%, 29 evaluable subjects per group, 87 in total, were required to show PK similarity. Accounting for possible dropouts, 35 subjects were to be included per group, 105 in total. Because equivalence can be claimed if only all pairwise comparisons are contained between the prespecified interval, no testing for multiplicity is required. The study has been designed to be analyzed in two independent submission dossiers in EU and in US, respectively. Therefore, the comparison of HD201 versus the respective reference products has been performed at an uncorrected level of significance and without calculating the power based on the two main comparisons. In addition, safety, tolerability, and immunogenicity data will be reported using descriptive statistics (arithmetic means, SD, CV%, min., max., and median).

### 2.5 Safety evaluations

The safety population consisted of all randomized subjects who had at least one dose of study drug. All adverse events (AEs) reported during the study were coded according to the Medical Dictionary for Regulatory Activities (version 21.1). Severity was graded as mild, moderate, and severe as defined in the protocol.

### 2.6 Immunogenicity evaluations

A total of five blood samples were collected for anti‐drug antibodies (ADA) detection and neutralizing antibodies (NAb): pre‐dose (0 h), 336 (Day 15), 672 (Day 29), 1008 (Day 43), and 1272 h (Day 54) post‐dose. ADA samples were analyzed using a validated immunoassay method with tiered approaches (screening, confirmatory, and titer assay) by Agilex Biolabs. Any confirmed positive ADA samples will have to be further tested for NAb using a validated cell‐based ADCC assay.

The detection of anti‐HD201 or anti‐trastuzumab antibodies in human serum is based on the bivalent characteristics of the antibody. During this incubation, anti‐HD201 or anti‐trastuzumab antibodies will bind to both the Sulfo‐tagged and biotinylated HD201 molecules to form an antibody complex bridge that will generate an electrochemiluminescent signal. The signal produced is proportional to the amount of anti‐ HD201 or anti‐trastuzumab antibodies present.

## 3 RESULTS

### 3.1 Subject characteristics and disposition

**FIGURE 1:**
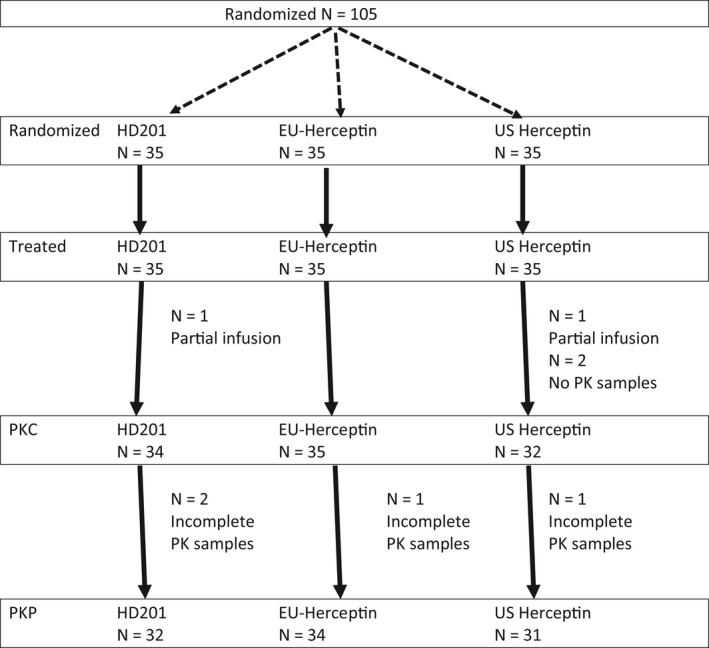
Subject disposition in TROIKA‐1 study. PKC population considered all subjects with PK samples collection. PKP population considered all subjects with full samples collection allowing the determination of PK parameters. N = number of subjects in the group ; n = number of subjects meeting specific criteria

A total of 105 healthy male subjects (35 in each arm) were randomized in this study and their baseline demographic characteristics were similar among the three treatment groups (Table 1). Figure 1 reports the distribution of the randomized subjects according to their completion of PK. All randomized subjects were included in safety and immunogenicity analysis. Serum trastuzumab concentrations summaries were based on the PKC population (HD201—34, EU‐Herceptin^®^—35, and US‐Herceptin^®^—32). Two subjects were prematurely withdraw due to infusion issues resulting in partial administration, PK samples were not collected due to venous access issues in two subjects which were also excluded from the PKC population. Pharmacokinetic parameter summary statistics and assessment of PK equivalence were performed on the PKP population (HD201—32, EU‐Herceptin^®^—34, and US‐Herceptin^®^—31). Three cases with missing PK samples were excluded from the PKP population. The incomplete collection was due to a lack of compliance without any reasons but in one case related to a fracture of the left thumb requiring surgery.

**TABLE 1:** Demographic data (safety population)

	HD201	EU‐Herceptin^®^	US‐Herceptin^®^	Overall
	*N* = 35	*N* = 35	*N* = 35	*N* = 105
Ethnicity	*n* (%)	*n* (%)	*n* (%)	*n* (%)
Hispanic or Latino	2 (5.7%)	3 (8.6%)	6 (17.1%)	11 (10.5%)
Not Hispanic or Latino	33 (94.3%)	32 (8.6%)	29 (82.9%)	94 (89.5%)
Race	*n* (%)	*n* (%)	*n* (%)	*n* (%)
Asian	5 (14.3%)	7 (20.0%)	6 (17.1%)	18 (17.1%)
Black or African American	—	1 (2.9%)	1 (2.9%)	2 (1.9%)
Native Hawaiian or other Pacific	—	1 (2.9%)	—	1 (1.0%)
White	29 (82.9%)	24 (68.6%)	23 (65.7%)	76 (72.4%)
Other	1 (2.9%)	2 (5.7%)	5 (14.3%)	8 (7.6%)
Age (years)				
Mean	27.5	29.6	29.7	28.9
SD	7.1	9.4	8.9	8.5
Median	25.0	28.0	27.0	27.0
Min–Max	19–49	18–54	19–52	18–54
Weight (kg)				
Mean	79.95	78.62	74.00	77.52
SD	11.41	11.69	9.59	11.13
Median	78.50	77.80	74.80	77.20
Min–max	59.6–110.6	60.4–109.1	55.8–92.1	55.8–110.6
Height (cm)				
Mean	178.80	179.53	176.87	178.40
SD	8.18	8.05	5.59	7.39
Median	177.00	178.00	177.00	178.00
Min–max	162.0–199.0	164.0–195.0	167.0–192.0	162.0–199.0
BMI (kg/m^2^)				
Mean	24.95	24.34	23.63	24.31
SD	2.73	2.83	2.62	2.76
Median	24.80	23.70	23.70	24.20
Min–max	19.9–29.3	20.3–30.0	19.0–29.2	19.0–30.0

Abbreviations: BMI, body mass index; Max, maximum; Min, minimum; *n*, number of subjects in the category; *N*, number of subjects in the population; SD, standard deviation.

### 3.2 PK results

**FIGURE 2:**
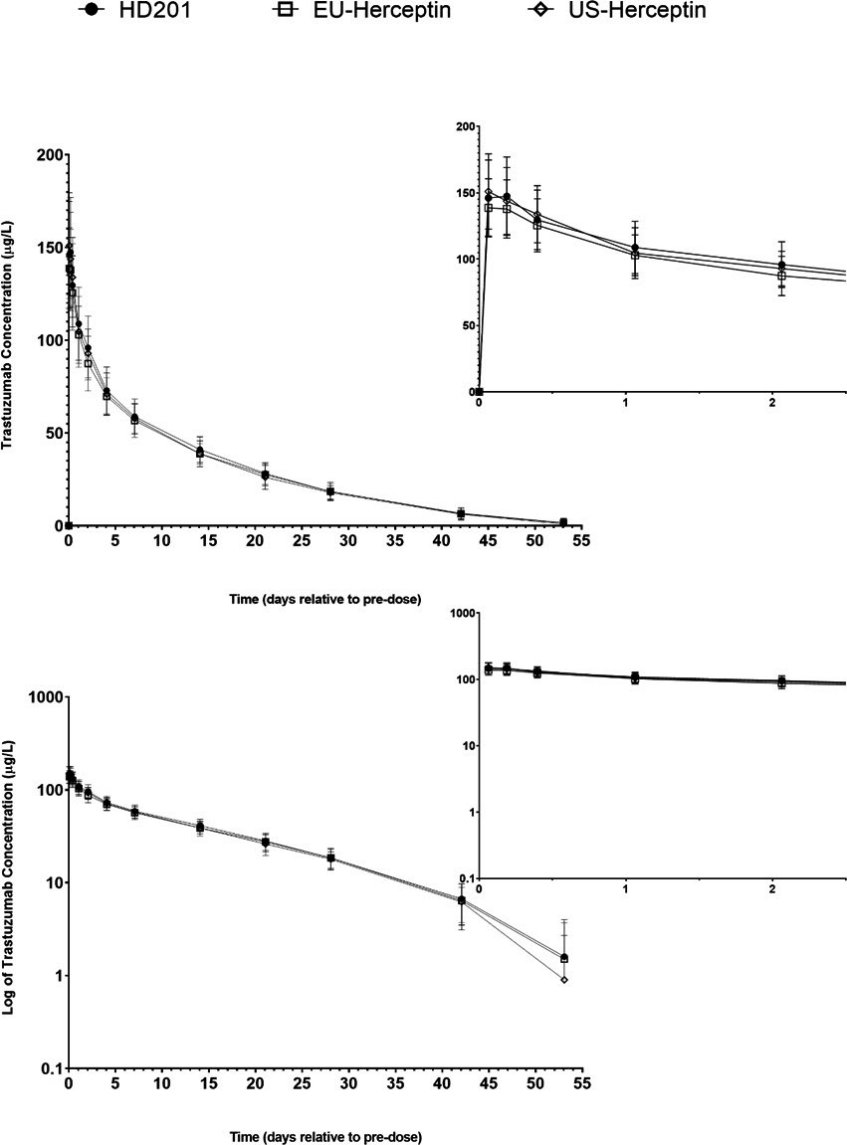
Trastuzumab serum concentration–time profiles (PKC population). Mean (±SD) trastuzumab concentrations over time are shown for all three groups on a linear scale (upper panel) and a semi‐logarithmic scale (lower panel). Insets show zoom of the first 48 hours after end of infusion. Number of subjects with serum concentrations reported at each time are provided in the source table. Note: Mean trastuzumab concentrations = 0.0 µg/L are not plotted on the semi‐logarithmic graph; some error bars are not shown on the semi‐logarithmic graph as negative values cannot be plotted logarithmically. The curves were based on the PKC concentration with 34, 35 and 32 subjects in the HD201, EU‐herceptin and US‐herceptin groups respectively

After administration of HD201, EU‐Herceptin^®^, or US‐Herceptin^®^, the percentage of the AUC_0–inf_ due to extrapolation (residual area) was 4.6%. 5.5%, and 4.5% of AUC_0–inf_. This indicates that the applied sampling schedule ensured the majority of AUC was captured and the range of times across which *K*_el_ was estimated was greater than twice the resultant *t*_1/2_. Nevertheless, there is one subject in the EU‐Herceptin group with 23% of residual area. Since less than 20% of the subjects had residual area <20%, no sensitivity analysis was performed in which this subject was excluded. All summarized PK parameters were therefore considered to be reliably estimated. The inter‐subject variability based on AUC_0–inf_ and AUC_0–last_, was characterized by a geometric CV ranging from 15.3% to 17.6%. For *C*_max_ the inter‐subject variability of EU‐Herceptin^®^ and US‐Herceptin^®^ were similar (15.5% and 16.6%) and slightly higher for HD201 (19.5%). PK profiles and PK parameters including AUC, *C*_max_, *t*_1/2,_ *V*_d_, Cl, and *T*_max_ were similar across treatment groups (Table 2; Figure 2).

**TABLE 2:** Summary of PK parameters for trastuzumab (PKP population)

Parameter	HD201 *N* = 32	EU‐Herceptin^®^ *N* = 34	US‐Herceptin^®^ *N* = 31
AUC_0–inf_ (h·µg/ml)			
Geometric mean	38 350	37 433	37 299
Geometric CV (%)	16.0	16.7	15.5
AUC_0–last_ (h·µg/ml)			
Geometric mean	36 588	35 337	35 620
Geometric CV (%)	17.6	17.0	15.3
*C*_max_ (µg/ml)			
Geometric mean	148.8	142.3	151.1
Geometric CV (%)	19.5	15.5	16.6
*t*_max_ (h)			
Median	1.7	3.2	1.6
Mean	2.9	3.6	3.3
SD	1.5	2.6	2.8
*t*_1/2el_ (h)			
Mean	234.2	243.1	238.5
SD	26.3	36.5	34.9
CV (%)	11.2	15.0	14.6
*K*_el_ (1/h)			
Geometric mean	297.7	288.0	293.6
Geometric CV (%)	11.1	14.2	14.2
CL (ml/h)			
Geometric mean	12.3	12.4	11.9
Geometric CV (%)	16.9	17.3	18.5
*V*_d_ (ml)			
Geometric mean	4133.3	4112.2	4056.2
Geometric CV (%)	17.3	19.8	18.7

Abbreviations: AUC_0–inf_, area under the concentration–time curve from time 0 extrapolated to infinity; AUC_0–last_, area under the concentration–time curve from time 0 to the last quantifiable data point; *C*_max_, maximum observed concentration; CV (%), coefficient of variation; Geo. CV (%), geometric coefficient of variation; Geo. Mean, geometric mean; *K*_el_, terminal elimination rate constant CL, systemic clearance; SD, standard deviation; *t*_1/2_, terminal half‐life; *t*_max_, time of maximum observed concentration; *V*_d_, volume of distribution.

Pairwise comparisons for PK endpoints (AUC_0–inf_, AUC_0–last_, and *C*_max)_ in all groups provided confidence intervals included between the prespecified equivalence margin (Table 3). Overall, systemic exposures, based on AUC_0–inf_, AUC_0–last_, and *C*_max_ after administration of HD201, EU‐Herceptin^®^, or US‐Herceptin^®^ were similar.

**TABLE 3:** Statistical analysis of PK parameters of HD201, EU‐Herceptin^®^, and US‐Herceptin^®^ (PKP population)

	Ratio (%) [90% CI]
HD201 (*N* = 32) vs. EU‐Herceptin^®^ (*N* = 34)	
AUC_0–inf_ (h·µg/ml)	
HD201/EU‐Herceptin^®^	102.45 [96.0; 109.4]
AUC_0–last_ (h·µg/ml)	
HD201/EU‐Herceptin^®^	103.5 [96.8; 110.8]
*C*_max_ (µg/ml)	
HD201/EU‐Herceptin^®^	104.6 [97.6; 112.2]
EU‐Herceptin^®^ (*N* = 34) vs. US‐Herceptin^®^ (*N* = 31)	
AUC_0–inf_ (h·µg/ml)	
EU‐/US‐Herceptin^®^	100.4 [93.9; 107.2]
AUC_0–last_ (h·µg/ml)	
EU‐/US‐Herceptin^®^	99.2 [92.7; 106.2]
*C*_max_ (µg/ml)	
EU‐/US‐Herceptin^®^	94.2 [87.8; 101.1]
HD201 (*N* = 32) vs. US‐Herceptin^®^ (*N* = 31) (FDA)	
AUC_0–inf_ (h·µg/ml)	
HD201/US‐Herceptin^®^	102.8 [96.2; 110.0]
AUC_0–last_ (h*µg/ml)	
HD201/US‐Herceptin^®^	102.7 [95.8; 110.1]
*C*_max_ (µg/ml)	
HD201/US‐Herceptin^®^	98.52 [91.7; 105.8]

Abbreviations: AUC_0–inf_, area under the concentration–time curve from 0 to infinity; AUC_0–last_, area under the concentration–time curve from 0 to last quantifiable analyte concentration; CI, confidence interval, *n*, number of subjects with the PK parameter; *C*_max_, maximum observed concentration; Mean, least squares mean.

### 3.3 Safety

Frequencies of subjects with TEAEs, treatment‐related TEAEs, and mild or moderate TEAEs were similar across the three treatment groups (Table 4). There were 124 adverse events in 65 subjects reported as related to study drug: 18 subjects (51.4%) in the HD201 treatment group, 23 subjects (65.7%) in the EU‐Herceptin^®^ group, and 24 subjects (68.6%) in the US‐Herceptin^®^ group. The frequency of subjects with TEAEs of special interest was lower in the HD201 group (20.0%) compared to the other treatment groups (EU‐Herceptin^®^: 34.3%; US‐Herceptin^®^: 31.4%). The most common adverse events of special interest related to treatment were infusion‐related reactions which occurred in 2 (5.7%), 7 (20.0%), and 11 (31.4%) subjects in the HD201, EU‐Herceptin^®^, and US ‐Herceptin^®^ group, respectively. No cardiac events were reported in the three treatment groups.

**TABLE 4:** Summary of treatment‐emergent adverse events

Subjects presenting with any:	HD201		EU‐Herceptin^®^		US‐Herceptin^®^	
	*N* = 35		*N* = 35		*N* = 35	
	*n*	%	*n*	%	*n*	%
TEAE	27	77.1	30	85.7	29	82.9
Treatment‐related TEAE	18	51.4	23	65.7	24	68.6
Treatment‐emergent SAE	–	–	1	2.9	–	–
Treatment‐related, treatment‐emergent SAE	–	–	–	–	–	–
TEAE of severity						
Mild	27	77.1	29	82.9	28	80.0
Moderate	1	2.9	9	25.7	6	17.1
Severe	–	–	–	–	–	–
TEAE leading to study discontinuation	–	–	–	–	–	–
TEAE of special interest	7	20.0	12	34.3	11	31.4

Analysis performed on the safety population; TEAE, treatment‐emergent adverse event; SAE, serious adverse event; *N*, number of subjects in the group; *n*, number of subjects with event.

### 3.4 Immunogenicity

One subject in the EU‐Herceptin^®^ group tested positive for anti‐drug antibody (ADA) at baseline (prior to dosing). This subject did not test positive at any time after receiving the study drug. No test for neutralizing antibodies was performed for this subject as ADA was detected prior to study drug administration.

## 4 DISCUSSION

The purpose of this study was to compare the pharmacokinetics (PK) of HD201, US‐Herceptin^®^, and EU‐Herceptin^®^ in healthy male subjects after intravenous administration of a single dose. This three‐arm parallel study design was chosen based on similar studies conducted for other trastuzumab biosimilar candidates, due to the long estimated half‐life of trastuzumab (approximately 12.6–26.6 days) and to avoid the potential influence of immunogenicity upon multiple dosing.^6^, ^7^, ^8^, ^9^, ^10^, ^11^ A single dose of 6 mg/kg was selected to demonstrate PK similarity in this study in accordance with the FDA guidelines.^3^ This dose was most likely to provide clinically meaningful and interpretable data as it represents the recommended maintenance dose of Herceptin for treatment. This study was conducted on healthy volunteers in accordance with EMA and FDA guidelines on similar biological medicinal products.^3^, ^4^ Additionally, only males were selected for this study since male volunteers to avoid the formation of neutralizing anti‐trastuzumab antibodies in women who are more likely to require trastuzumab for the treatment of breast cancer at some point in their life.

EAGLE‐I‐12 Phase I study was conducted in 2012 with more than sufficient coverage of theoretical drug clearance to make sure that the PK comparison can be obtained between reference herceptin and HD201. Over the last decade, many scientific evidence have been compiled to prove that PK similarities can be fully assessed with a lesser duration of profiling. A shortened PK sampling day was considered in TROIKA‐1 study as other trastuzumab biosimilar phase I studies. Consequently, the considered *C*_max_ in this study did not represent a stringent determination of this parameter but the concentration provided in the sample collected at 1.5 h. This rounded value appeared acceptable because the main objective was to perform a pairwise comparisons of PK and not to characterize the PK profile of trastuzumab.

Overall exposure to trastuzumab, assessed by AUC_0–inf_, AUC_0–last_, and peak systemic exposure to trastuzumab assessed by *C*_max_, was shown to be comparable after administration of HD201 and EU‐Herceptin^®^, and after administration of HD201 and US‐Herceptin^®^. For both comparisons, the 90% CIs for the ratio of the geometric mean of AUC_0–inf_, AUC_0–last_, and *C*_max_, were all contained within the prespecified margin of 80.00% to 125.00%. In addition, the US‐Herceptin^®^ and EU‐Herceptin^®^ were equivalent based on a similar comparison process. All pairwise comparison was included in the prespecified margins then the PK equivalence can be stated between HD201, US‐Herceptin^®^, and EU‐Herceptin^®^. In addition, the other secondary PK parameters including residual area, *t*_max_, *t*_½ el_, *K*_el_, Cl, and *V*_d_ were similar between the three treatment groups.

The secondary objectives of this study were to assess the safety, tolerability, and immunogenicity of HD201 and the EU and US reference products Herceptin^®^. A total of 86 subjects had adverse events, 27 (77.1%) in the HD201 group, 30 (85.7%) in the EU‐Herceptin^®^ group, and 29 (82.9%) in the US‐Herceptin^®^ group. A majority of adverse events were mild in severity. There were 33 adverse events of special interest in 30 subjects. A majority of adverse events of special interest were “Infusion‐related reactions,” and were reported in a lower frequency in subjects in the HD201 treatment group compared to subjects in the other two treatment groups. No reason could specifically explain this unbalanced distribution of infusion‐related reactions. Other adverse events of special interest were general disorder and administration site conditions (Chest pain, Pyrexia, and Influenza‐like illness) and occurred more frequently in the HD201 (3 subjects; 1 (2.9%) event each, 8.6%) and EU‐Herceptin^®^ (3 subjects; 1 (2.9%) event each, 8.6%) treatment groups than in the US‐Herceptin^®^ treatment groups (0 subjects). There were no notable differences between the three treatment groups in electrocardiograms, echocardiograms, clinical laboratory evaluations, and vital signs. One subject in the EU‐Herceptin^®^ group tested positive for anti‐drug antibody (ADA) at baseline prior to study drug administration. This subject did not test positive at any time after receiving the study drug. Therefore, no test for neutralizing antibodies (NAb) was performed.

Overall, HD201 demonstrates equivalent PK to both EU‐Herceptin^®^ and US‐Herceptin^®^ following a single i.v. infusion of 6 mg/kg over 90 min. TROIKA‐1 study is consistent with the EAGLE‐I‐12 phase I study findings and established that manufacturing process changes for HD201 did not impact the safety and PK equivalence to the reference product. A phase III randomized study (TROIKA) in patients with HER2+ early breast cancer is ongoing (NCT03013504). TROIKA and TROIKA‐1 study utilize the same HD201 batches from the same manufacturing process. This phase III study is aimed to demonstrate a similar activity in neoadjuvant setting for early breast cancer as previously demonstrated by other biosimilar trastuzumab candidates.^12^, ^13^, ^14^ The TROIKA study represents the ultimate step of development before a submission to regulatory agencies for commercial distribution. Similarly to other trastuzumab biosimilars, a secondary PK assessment is conducted in the phase III randomized TROIKA study to confirm the equivalence of HD201 versus the reference trastuzumab in patients treated by multiple consecutive cycles of treatment.^12^, ^13^, ^14^, ^15^

## DISCLOSURE

Martin Demarchi, Pierre Coliat, Xavier Pivot, Kristi Mclendon, and Alexandre Detappe do not have any conflicts of interest for this article.

Jocelyn Chung Shii Hii, Peggy Feyaerts, Felicia Ang, and Litha Jaison were employees of Prestige Biopharma Ltd Singapore Michael Jinwoo Kim, Lisa Soyeon Park, were employees of Prestige Biopharma Ltd Singapore and Prestige Biologics Co Ltd, Korea.

Marie Paule Derde and Filip Deforce were employees by DICE Ltd which had a memorandum of understanding with Prestige Biopharma Ltd.

## AUTHORS’ CONTRIBUTIONS

XP, LSP, PF, MJK, FD, and LJ had substantial contributions to the conception and design of the work; KM, PF, FA, and JCSH had substantial contribution to the acquisition, MPD and FD performed the analysis, XP, MD, PC, and AD performed the interpretation of data for the work; MD, MPD, and XP Draft the work.

PC, KM, JCSH, PF, F A, LJ, FD, MJK, LSP, and AD revise the manuscript critically for important intellectual content.

All author provided a final approval of the version to be published; AND agree to be accountable for all aspects of the work in ensuring that questions related to the accuracy or integrity of any part of the work are appropriately investigated and resolved.

## DATA QUALITY ASSURANCE

The clinical site has established Quality Control (QC) and Quality Assurance (QA) systems with written SOPs to ensure that the study was conducted and data were generated, recorded, and reported in compliance with the protocol, GCP, and applicable regulatory requirements. A rigorous QC program was applied to ensure the accuracy of all data and reports.

In accordance with the principles of GCP and GLP, the study could be inspected by the QA unit of the clinical sites, regulatory authorities, the Sponsor and Syneos Health. The Sponsor was entitled to access information.

## ROLE OF THE FUNDING SOURCE

The funding source validated the study as designed by the trial's steering committee as well as subsequent amendments. The sponsor organized the collection of data. Data were analyzed by DICE. Data were interpreted by the trial's steering committee including XP, MD, PC, FD, PF, MPD, and AD independently from the sponsor. The corresponding author had full access to all of the data and had the final responsibility to submit for publication.

## Data Availability

Data collected for the study, including individual participant data and a data dictionary defining each field in the set, will be made available to others; all available data will be de‐identified participant data. The study protocol, statistical analysis plan, informed consent form, and ethics committee approval are available. To access the data, a request should be submitted at Prestige Biopharma with a scientific proposal including objectives. The data will be shared after approval and by the steering committee of the trial. Approval by the ethics committee might be required according to the type of proposal and objectives.
